# Not that many leech species after all: *Myzobdella lugubris* and *Myzobdella patzcuarensis* (Annelida: Hirudinida) are the same species

**DOI:** 10.1007/s11230-024-10160-5

**Published:** 2024-05-03

**Authors:** Gerardo Torres-Carrera, Yanet Velázquez-Urrieta, Ana Santacruz

**Affiliations:** 1 Posgrado en Ciencias Biológicas, Universidad Nacional Autónoma de México, 04510 Mexico, Mexico; 2 Laboratorio de Helmintología, Departamento de Zoología, Instituto de Biología, Universidad Nacional Autónoma de México, 04510 Mexico, Mexico; 3 Present Address: Stowers Institute for Medical Research, Kansas City, MO USA

## Abstract

The genus *Myzobdella* groups five species of leeches parasites of fishes mainly of freshwater but with tolerance to brackish waters. Native distribution of these species includes the New World from North to South America. *Myzobdella lugubris* Leidy, 1851, the type species of the genus, was briefly described based on specimens from the USA, but subsequently their morphology, known distribution and host range were expanded; however, less is known about the other four species of the genus. As part of a survey focusing on characterizing the diversity of leeches from Mexico, specimens of *Myzobdella patzcuarensis* (Caballero, 1940), from the type locality of the species were included for the first time in a phylogenetic study. In addition, specimens assigned to *Myzobdella* from the southeast of Mexico as well as from Nicaragua, were also included. In the resulting phylogenetic tree, our newly generated sequences were found nested in the same clade that *M. lugubris*; with unresolved relationships and relatively low genetic divergence, suggesting conspecificity. In addition, the internal morphology of the specimens of *Myzobdella* from Mexico is consistent with the description of *M. lugubris*. Our morphological examination reveals high degrees of variability in the external pigmentation of the specimens. Based on our results we formally synonymize *M. patzcuarensis* under *M. lugubris*.

## Introduction

Piscicolidae Johnston 1865 (Annelida: Hirudinida) is a family of parasitic leeches mainly associated with fishes, with about 170 described species around the world, two-thirds of which are marine species (Magalhães et al., 2021). Investigation about the systematics of this group is scarce in comparison with their freshwater counterparts, and the classification within the family remains unresolved (Williams & Burreson, 2006). Historically, three subfamilies were recognized: Platybdellinae Epshtein 1970, Piscicolinae Johnston 1865 and Pontobdellinae Llewellyn 1966. These subfamilies were proposed in the basis on the absence or presence of a single, or two pairs of pulsatile vesicles, respectively. So far, only members of Pontobdellinae have been recovered as a monophyletic group in phylogenetic studies (Williams & Burreson, 2006; Utevsky & Utevsky, 2018).

The genus *Myzobdella* was erected by Leidy, 1851 based on the study of specimens found attached to crabs of the genus *Callinectes* Stimpson (Decapoda: Portunidae) from an undefined locality, but must likely from Philadelphia, USA. The description of *M. lugubris*, the type species of the genus, is brief and provides no information about its geographic distribution or the designation of the holotype or paratypes; however, it was thought to be a brackish species (Leidy & Cassin, 1850). Meyer (1940) recognized the genus *Illinobdella* to include four freshwater fish-leech species from North America: *Illinobdella alba* Meyer, 1940, *I. elongata* Meyer, 1940, *I. moorei* Meyer, 1940 and *I. richardsonii* Meyer, 1940 and differentiated the new species from *M. lugubris* based on their habitat and host association, since all *Illinobdella* species are typically found in freshwater environments parasitizing fishes. In the same year, a morphologically similar species was described in Mexico: *Illinobdella patzcuarensis*; parasite of the charal fish *Chirostoma estor* Jordan (Teleostei: Atherinopsidae) from Patzcuaro Lake, Michoacán.

Further studies on *Myzobdella lugubris* have established that this species has a broad geographic distribution along USA and Canada, dwelling brackish and freshwater habitats, and interestingly, able to parasitize both crustaceans and fish (see Moore, 1946; Hutton & Sogandares-Bernal, 1959; Klemm, 1972; Daniels & Sawyer, 1975). Sawyer et al. (1975) and Daniels & Sawyer (1975) found that neither habitat nor host preference are reliable features for the separation of species of *Illinobdella* and *M. lugubris*. Also, such studies found no differences in internal and external anatomical traits between species, therefore all* Illinobdella* species from USA were transferred to *Myzobdella* and synonymized with *Myzobdella lugubris,* with the notable exception of *M. patzcuarensis*. Currently, in addition to *M. lugubris* and *M. patzcuarensis*, three species are considered valid within the genus: *Myzobdella reducta* (=*Piscicolaria reducta* Meyer, 1940), from USA and *Myzobdella uruguayensis* Mañé-Garzón and Montero, 1977 and *Myzobdella platensis* (Cordero, 1933) from South America (Uruguay, Argentina and Brazil). Previous phylogenetic analysis of Piscicolidae included only *M. lugubris* and *M. reducta* (Williams & Burreson, 2006), showing low genetic variation between both species; hence, Saglam et al. (2018) suggested that both specimens might belong to *M. lugubris*, so the remaining species are unrepresented.

Records of *Myzobdella lugubris* have been reported in Hawaii and Italy (Williams & Burreson, 2006; Liuzzo et al., 2018), where they were likely accidentally introduced (Lages et al., 2021). More recently, *Myzobdella* sp. was also reported in Nicaragua, parasitizing freshwater cichlids (Santacruz et al., 2022). *Myzobdella* species are important for the economy due to the damage they may cause to fish (Appy & Cone, 1982; Faisal et al., 2011; Volonterio et al., 2004) and crab hosts (Severino-Rodrigues & de Almeida-Duarte, 2020; Zara et al., 2009); and due their potentially role as vectors of blood parasites (Faisal & Schulz, 2009).

As part of a survey to characterize the morphologic and genetic diversity of fish leeches from Mexico, we collected samples of *M. patzcuarensis* from its type locality (Lake Patzcauro, Mexico) and additional specimens from the states of Veracruz, Yucatán, Quintana Roo, Mexico and from Nicaragua lake, Nicaragua.

## Materials and methods

### Sample collection

Fish specimens of *Vieja* sp. were recollected in January–February 2019, and November 2021 in Escondida lagoon, Los Tuxtlas, Veracruz, Mexico (18° 35′ 25″ N, 95° 05′ 23″ W). Fish specimens were captured using cast net and transported alive to the laboratory. Fish were examined for leeches attached to their external surface under a stereomicroscope. Leeches were removed with fine brushes and placed in tap water and examined *in vivo*, to distinguish morphological traits. For further morphological studies, leeches were fixed in warm (near boiling) 4% formalin and preserved in 70% ethanol. For molecular analyses, three specimens were preserved in 100% ethanol. Additionally, we generated DNA sequences of three specimens from Patzcuaro, Michoacan, Mexico (19° 37′ 30″ N, 10° 37′ 31″ W), donated by A. Oceguera-Figueroa; two specimens from Sisal, Yucatán (21° 11′ 29.6″ N, 89° 56′ 53.9″ W) and Isla Tamalcab, Quintana Roo, Mexico (18° 36′ 29″ N, 88° 12′ 14″ W), donated by J. Arroyave and S. Monks respectively; and two specimens preserved in alcohol and deposited in the Colección Nacional de Helmintos (CNHE), Instituto de Biología, Universidad Nacional Autónoma de México (UNAM) under number: 7252, of *Myzobdella* sp. collected in Nicaragua lake, Nicaragua (11° 10′ 05.23″ N, 84° 58′ 44.08″ W) (Santacruz et al. 2022).

### Morphological analyses

Four leeches were stained with Gomori’s trichrome, and nine with a mixture of Meyer’s paracarmin and haematoxylin, dehydrated through graded ethanol series, cleared in methyl salicylate, and mounted in Canada balsam. Morphometric data were obtained with a micrometric ruler adapted to a light microscope Olympus XI (Olympus, Tokyo, Japan). Measurements are presented in in mm as the mean with the range and number of individuals studied in parentheses. For Scanning electron microscopy (SEM), specimens were dehydrated through ethanol series, dried using a K850 Critical Point Drier (Emitech, Ashford, England), sputter coated with gold with Q150R modular Coating System (Ashford, England), and examined at 15 kV in a Hitachi SU1015 SEM (Hitachi, Tokyo, Japan). Specimens collected were compared with voucher specimens of *Myzobdella patzcuarensis* deposited at the Colección Nacional de Helmintos (CNHE), Instituto de Biología, Universidad Nacional Autónoma de México (IB-UNAM). Newly collected specimens were deposited in CNHE.

Morphological identification of specimens was based on Sawyer et al. (1975) and Saglam et al. (2018); the diagnostic traits which bear *Myzobdella lugubris* are: one pair of eyespots, tegument translucent, trachelosome and urosome visibly divided, two annuli separating gonopores, five pairs of testisacs and midbody somites with 12 or 14 annuli.

### Molecular procedures

Tissue from the leech caudal sucker was removed and used for total DNA extraction using Invitrogen PureLink® Genomic DNA mini kit (ThermoFisher Scientific, Pittsburgh, Pennsylvania) following the manufacturer’s protocol. The next mitochondrial loci: cytochrome C oxidase subunit 1 (*COI*), nicotinamide adenine dinucleotide dehydrogenase subunit I (*nad1*), *12S* rDNA and the nuclear: *18S* rDNA, were amplified following Williams & Burreson (2006) and Saglam et al. (2018) protocols. PCR products were visualized by electrophoresis on an agarose gel. Successful amplifications were purified using CentriSep 96 filter plates (ThermoFisher Scientific, Pittsburgh, Pennsylvania) with Sephadex G-50 (Cytiva, Marlborough, Massachusetts). Sequencing reactions included 0.4 µl BigDye Terminator v. 3.1 (Applied Biosystems, Waltham, Massachusetts), 2 µl Buffer 5x, 4 µl ddH_2_O, 1 µl of primer at 10 µM, and 3 µl purified PCR product (total volume 10 µl). Samples were purified using Sephadex G-50, then 25 µl de EDTA 0.5 mM was added to each sample and finally sequenced in an ABI-PRISM 3100 (Applied Biosystems® Waltham, Massachusetts) sequencer at the Laboratorio Nacional de Biodiversidad (LANABIO), IB-UNAM. Complementary sequences were assembled and edited using Geneious ver. 5.1.7 (Biomatters Ltd., Auckland, New Zealand).

### Phylogenetic analysis

Multiple sequence alignment was performed in MAFFT online version (Katoh et al., 2018) applying default parameters. *Pontobdella macrothela* Schmarda, 1861 was used as outgroup based on previous phylogenetic analyses (Saglam et al., 2018; Utevsky & Trontelj, 2004). Details of the sequences included in the analysis are provided in Table 1. Concatenated matrix including the four genetic markers was constructed with Mesquite v. 3.7 (Maddison & Maddison, 2023). Final dataset included 23 terminals and 3317 aligned characters. Phylogenetic analysis was performed under Maximum Likelihood approach in IQ-Tree (Nguyen et al., 2015) using the concatenated dataset, with evolutionary model found with ModelFinder (Kalyaanamoorthy et al., 2017) indicating each partition in a file apart, called with -p function and with rapid Bootstrap option (Hoang et al., 2017) with 1000 pseudo-replicates. Best model fit to *cox1* was TIM+F+G4*,* to *nad1* K3Pu+F+G4, to *12S* K3Pu+F and to *18S* was TNe+I. We trimmed *cox1* dataset to 576 bp to minimize alignment gaps and calculated pairwise genetic distances with Mesquite v. 3.7 under the K2P substitution model, as is suggested by Nei & Kumar (2000).

**Table 1:** Species, localities, and sequences GenBank accession numbers of family Piscicolidae included in our analysis. New sequences are in bold.

Taxon	Locality	GenBank accession number				
		*cox1*	*nad1*	*18S*	*12S*	Source
Pontobdellinae						
*Pontobdella macrothela*	Heron Island, Australia	DQ414340	DQ414385	DQ414295	AY425440	Williams & Burreson (2006)
Piscicolinae						
*Piscicola geometra*	France	AF003280	AY047334	AF115995	AF099959	Siddall & Burreson (1998), Apakupakul et al. (1999), Light & Siddall (1999)
Platybdellinae						
*Austrobdella californiana*	California, USA	DQ414304	DQ414349	DQ414258	–	Williams & Burreson (2006)
*Myzobdella lugubris*	Virginia, USA	DQ414324, DQ414323, AF003269	DQ414368, DQ414367, AY047332	DQ414278, AF115994, DQ414277	–	Siddall & Burreson (1998), Williams & Burreson (2006)
*Myzobdella lugubris*	Hawaii, USA	DQ414325	DQ414369	DQ414279	–	Williams & Burreson (2006)
*Myzobdella lugubris*	Le Cesine, Italy	MG820612	–	–	–	Liuzzo et al. (2018)
*Myzobdella lugubris*	Pennsylvania, USA	KY440059, KY440058	–	KY440056	KY440057	Saglam et al. (2018)
*Myzobdella lugubris*	Maryland, USA	KU905914, KU905849	–	–	–	Aguilar et al. (Unpublished)
*Myzobdella lugubris* (=*Myzobdella patzcuarensis*)	Michoacan, Mexico	**OQ659461**, **OQ659462**, **OQ659463**	–	–	**OQ675141**, **OQ675142**	**This study**
*Myzobdella* sp.	Veracruz, Mexico	**OQ659464**	**OR508994**	**OQ659394**	**OQ675143**	**This study**
*Myzobdella* sp.	Veracruz, Mexico	–	**OR508995**	–	**OQ675144**	**This study**
*Myzobdella* sp.	Veracruz, Mexico	**OQ659465**	**OR508993**	**OQ659393**	**OQ675145**	**This study**
*Myzobdella* sp.	Yucatan, Mexico	**OQ659466**	–	–	–	**This study**
*Myzobdella* sp.	Quintana Roo, Mexico	**OR511726**				**This study**
*Myzobdella* sp.	Nicaragua Lake, Nicaragua	**PP434471, PP434472**				**This study**
*Myzobdella reducta*	Tennessee, USA	DQ414339	DQ414384	DQ414294		Williams & Burreson (2006)

## Results

### Phylogeny

**Fig. 1:**
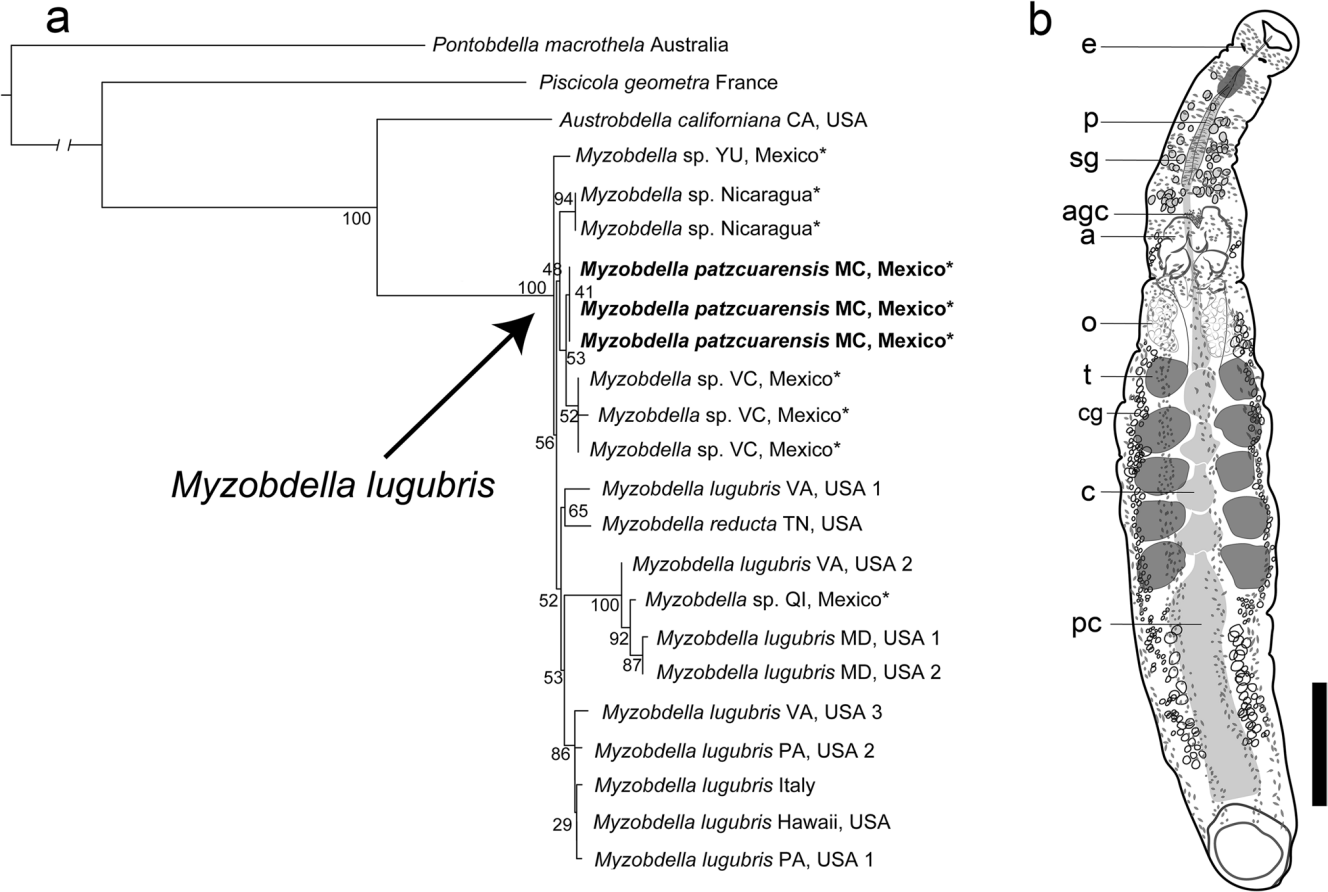
a Maximum likelihood tree based on concatenate dataset, showing the phylogenetic position of *Myzobdella patzcuarensis* sequences from the type locality in bold, new sequences signalized with an asterisk. b Drawing of internal anatomy of *Myzobdella lugubris* collected in Veracruz, Mexico CNHE: 11660. (e) Eyespot; (p) proboscis; (sg) salivary glands; (agc) accessory gland cells; (a) atrium; (o) ovisac; (t) testisacs; (cg) cocoon glands; (c) crop; (pc) post caeca. Scale bar 1 mm.

In the resulting phylogenetic tree, the genus *Myzobdella* is recovered as a monophyletic group with high node support (Fig. 1a). The relationships between samples of *M. lugubris*, *M. patzcuarensis* and *Myzobdella* sp. from Yucatán and Veracruz and Nicaragua are unresolved with marginal branch lengths, and nodes poorly supported. There was no noticeable phylogenetic separation between samples from Mexico and Central America with those from the USA and Italy.

The *cox1* mean genetic distance between *M. patzcuarensis* from its type locality and *Myzobdella* sp. from Veracruz, Yucatán and Quintana Roo, Mexico is 1.16% (0 to 2.47) and between *M. patzcuarensis* and *Myzobdella* sp. from Nicaragua is 0.52% (0.52). Instead, between *M. patzcuarensis* and *M. lugubris* from USA is 1.4 (0.69 to 2.47). *Myzobdella lugubris* from the USA and Italy exhibit interpopulation divergences ranging from 0.17% to 2.86% and new collected samples fit in this interval. The sample from Quintana Roo is nested with *M. lugubris* from VA, USA (1) and MD, USA (1, 2) (Fig. 1a). This sample has pairwise genetic distances that do not surpass 2.7% with respect to the remaining samples from Mexico and from Nicaragua. The mean genetic distances within *Myzobdella* clade are 1.35% (0 to 3.01).

### Morphology

Family Piscicolidae Johnston, 1865

*Myzobdella* Leidy, 1851

*Myzobdella lugubris* Leidy, 1851

*Myzobdella patzcuarensis* (Caballero, 1940) **n. syn.**

Zoobank identifier: urn:lsid:zoobank.org:act:6B4B6675-A0CF-4FD7-9F7A-FF49B9E8E154

*Other synonyms*: *Ichthyobdella funduli* Verrill, 1872; *Piscicola funduli* Pratt, 1935; *Illinobdella alba* Meyer, 1940; *I*. *elongata* Meyer, 1940; *I. richardsoni* Meyer, 1940; *I. moorei* Meyer, 1940; *Myzobdella lubrigis* Pearse, 1948; *M. funduli* Moore, 1952; *M. moorei* Meyer & Moore, 1954; *Ichthyobdella rapax* Wass, 1972.

*Collection material from CNHE*: *Myzobdella patzcuarensis* from its type locality Patzcuaro, Michoacán, five specimens (CNHE: 1684, 1692, 1684) ex. *Algansea lacustris* Steindachner; two specimens (CNHE:1687) ex. *Chirostoma estor*; two specimens (CNHE: 1688, 1690) ex. *Goodea atripinnis* Jordan*. Myzobdella patzcuarensis* from Infiernillo, Michoacán, one specimen (CNHE: 673) ex. *Oreochromis aureus* (Steindachner) and one (CNHE: 1699) ex *Coptodon zillii* (Gervais), one specimen (CNHE: 1700) ex. *Ictalurus balsanus* (Jordan et Snyder). *Myzobdella* sp. two specimens (CNHE: 7252) from Lake Nicaragua, ex *Parachromis* sp., caudal sucker processed to molecular analyses.

*Newly collected samples*: 25 specimens from Los Tuxtlas, Veracruz parasitizing the pectoral fin of *Vieja* sp. Nine stained individuals (CNHE: 11660); two individuals SEM processed, three individuals aimed to molecular analyses and 11 alcohol preserved specimens (CNHE: 11661).

*Additional material observed*: paratype of *Illinobdella alba* from Illinois, USA ex. *Ictalurus punctatus* (Rafinesque) collected by M. C., Meyer, 1936 (CNHE: 1702).

**Fig. 2:**
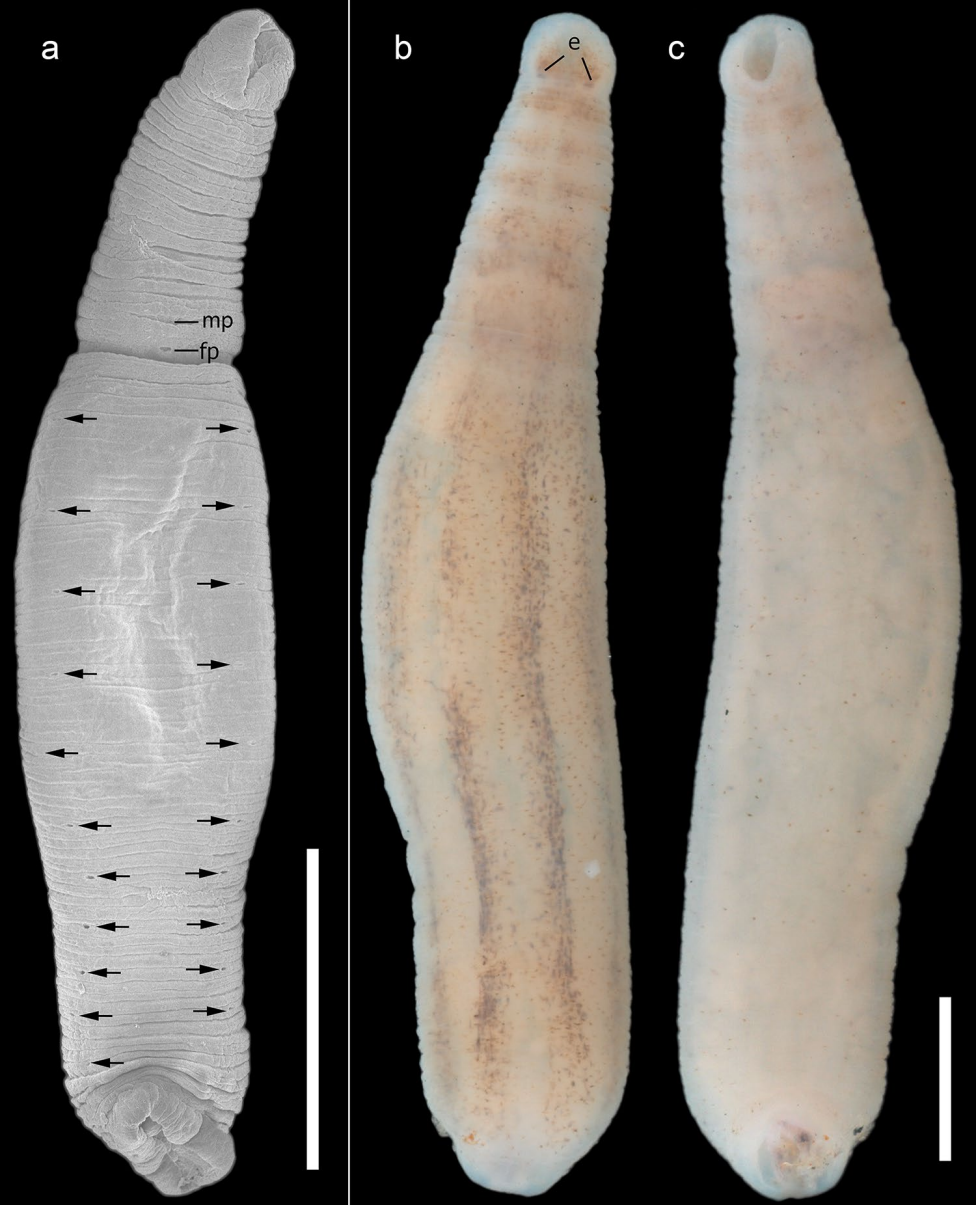
*Myzobdella lugubris* from Los Tuxtlas, Veracruz. a SEM microphotograph of ventral surface; nephridiopores are indicated with black arrows; male gonopore (MP) and female gonopore (FP) are indicated with black lines. b Dorsal and c and ventral views. (e) Eyespots. Scale bar 1 mm

*External morphology*. Body elongated and smooth, 10.2 total length (4–20, n = 24), 1.6 of maximum width (0.8–3, n = 24); divided in two portions, trachelosome and urosome. Integument translucent. Anterior sucker small, semi-circular 0.6 length (0.40–0.85, n = 24) and 0.7 wide (0.45–0.8, n = 24). Nuchal constriction separating anterior sucker present. Mouth pore small, located in the center of the anterior sucker. Eyespots visible, one pair at somite III. Dorsal surface of the trachelosome with seven transversal stripes, first stripe beginning anterior to the eyespots and the last just before urosome (Figs. 1b, 2b, c). Constriction separating trachelosome and urosome conspicuous. Urosome with three to four longitudinal stripes. Gonopores on the ventral surface of the urosome, separated by two annuli, XI a2 and XI a3 a1 (Fig. 2a). Midbody somites with 14 annuli. Nephridiopores of the trachelosome not seen. Eleven pairs of nephridiopores visible at ventral surface of urosome, situated on lateral margin of the ventral surface (Fig. 2a). Posterior sucker smaller than the maximum body wide 0.98 length (0.58–1.2, n=24) and 1 wide (0.59–1.9, n = 24).

*Internal morphology*. Pharynx short, reaching somite X. Salivary glands abundant and diffused in parenchyma in somites IX to X or reaching XI, apparently with conducts opening to the base of proboscis. Bacteriomes not seen. Crop caeca with five pairs of globular chambers, located between testisacs. Post caecum saccular, almost reaching posterior end of body. Intestinal caeca not seen. Male reproductive system consisting in five pairs of testisacs. Testisacs large and oval, intersegmentally arranged. Accessory gland cells present, compact, anterior to atrial cornua. Atrial cornua voluminous, tubular not coiled, embracing until 2/3 of trachelosome maximum wide. Deferent conducts coiled. Female reproductive system consisting in a pair of ovisacs, tubular, long and s-shaped, reaching the first pair of testisacs. Cocoon glands lateral, with a longitudinal path, from clitellar area to level of the post-caecum.

### Remarks

Caballero (1940) described *Illinobdella patzcuarensis* based on specimens from Lake Pátzcuaro, Michoacán. In spite of clearly recognizing the similarities between the new species and *Illiobdella alba*, described from the USA, the author highlighted some morphological differences of the Mexican forms, such as the presence of 14 pairs of nephridiopores and differences in the shape of the atrium (not specified) and mainly the presence of “reserve cells” in *M. patzcuarensis*. Based on the specimens studied here, “reserve cells” located along the proboscis (Fig. 1b) correspond to salivary glands, as described by Meyer in the descriptions of *Illiobdella* spp. Caballero found similar “reserve cells” along at the intestinal portion of body, reinforcing his idea of storage function. In the specimens studied here, these cells were found forming an antero-posterior path, laterally in the body. This type of cells were mentioned by Meyer in the description of *I. elongata* and *I. moorei* but he thought they were related with cocoon production. Probably this is the correct function of these cells, which are also present in other piscicolids and their occurrence depends on reproductive stage of the leech (Sawyer, 1986). Both *M*. *lugubris* from USA and those *Myzobdella* samples from Mexico have salivary glands of varying in size and some individuals present developed cocoon glands, whence, it is not a distinctive character between these taxa.

In the specimens studied here, 11 pairs of nephridial pores restricted to the urosome were observed; this number is consistent with description *I. alba* of Meyer (1940). In the transversal sections of *M. patzcuarensis* analyzed by Caballero, he found fourteen pairs of nephridiopores in the whole body (trachelosome and urosome). Thus, it is possible that the observations of Meyer (1940) were based only on the urosome (as in this study), and Caballero (1940) observed the remaining three pores situated at trachelosome. Three nephridial pores at trachelosome and eleven at urosome is consistent with the fourteen nephridial pores seen in Rhynchobdellida (see Sawyer, 1986, Vol. 1, Fig. 4.2).

Based on the current morphological evidence, no significant differences were found between *M. patzcuarensis* and *M. lugubris*. Therefore, we formally synonymize the former with *M. lugubris* – the valid name for the Mexican forms. Regarding the remaining internal traits, specimens assigned to *Myzobdella lugubris* from all known populations, both previously published, and this study have similar internal configuration. However, some differences in the external morphology between Mexican specimens and those from USA and Italy are remarkable. In the case of individuals from Patzcuaro, the body coloration has been reported as completely white (Caballero, 1940), while living individuals from Los Tuxtlas display black coloration with dark-brown to black stripes (Fig. 2b). The stripes in specimens from Los Tuxtlas differ from those of *M. lugubris* from Philadelphia, which were described as zig-zagged longitudinal stripes (Saglam et al., 2018), versus straight stripes in material examined here (Fig. 2b). This highlights the great variability in external pigmentation.

*Myzobdella lugubris* can clearly be differentiated from *M. platensis* and *M. uruguayensis* but not so from *M. reducta*. *Myzobdella lugubris* has a posterior sucker less width than body width, while in *M. platensis* posterior sucker is up to twice wider than body; *M. lugubris* has a pair of eyespots while *M. uruguayensis* presents two pairs. In the original description of* M. reducta* (=*Piscicolaria reducta*), mid-body somites are reported with three annuli and a total of eleven pairs of nephridiopores along the body, such characters were used to distinguish *M. reducta* from other species (Meyer, 1940); however, this number of annuli per somite might be wrong given that the most common condition for the genus is 14 annuli per complete somite (Moore, 1946; Sawyer, 1986; Mañé-Garzón & Montero, 1977), and common in other Piscicolidae. For example, in *Austrobdella*, the sister group of *Myzobdella* (Williams & Burreson, 2006; Utevsky et al., 2021) has six annuli per somite (Burreson, 1977; Curran et al., 2016). Body coloration has been suggested as diagnostic character to differentiate between *M*. *lugubris* and *M. reducta*, however, given the variation in body coloration, the separation between both and the validity of the last mentioned requires further analysis.

Moore (1938) recorded a leech morphologically similar to *Myzobdella platense* (=*Piscicola platense*) parasitizing *Rhamdia guatemalensis* in a cave (cenote) in Yucatán, México. Its identification was based mainly on the characteristics of the clitellum, however, the size of posterior sucker (1 mm), with respect to maximum width of body (1.2 mm) resembles *M. lugubris* instead of *M. platensis* (see above). In addition, the specimens were poorly preserved, so the identification is doubtful (Moore, 1938). In our phylogenetic tree, the leech specimen from Yucatán, was found nested within the clade comprising *M. lugubris* sequences, suggesting that samples from the area may be conspecific with *M. lugubris*.

## Discussion

*Myzobdella patzcuarensis*, as well as their host, *Chirostoma estor* (‘pescado blanco’) have been considered part of the representative fauna of Lake Patzcuaro, Mexico (Oceguera-Figueroa & León-Règagnon, 2014; Torres, 1993). According to the new data presented here, *M. patzcuarensis* seems to be a junior synonym of *M. lugubris* instead of representing an independent species. Historical records indicate that in 1930, the largemouth bass *Micropterus salmoides* (Lacépède) was introduced to Lake Patzcuaro with fishery purposes (Solorzano, 1955), and likely *M. lugubris* was co-introduced.

Largemouth bass were also introduced to Nicaragua between 1950 and 1960 but their populations disappeared years later (Heidinger, 1976); the introduction of *M. lugubris* could have been co-introduced in the area and then colonized native fish. Alternatively, the introduction of this leech could be recent since this species has been recorded only in a single *Parachromis* sp. individual from Lake Nicaragua (Santacruz et al., 2022). Additional records of introduced leeches in South and Central America have been published before, *M. lugubris* has been reported in Venezuela (Heidinger, 1976) and more recently, Oceguera-Figueroa & Pacheco-Chaves (2012) recorded the Asian leech *Barbronia weberi* in Costa Rica and Kvist et al. (2018) recorded *Placobdella parasitica* in Panama.

Based on the present work, with the synonymization of *M. patzcuarensis* with *Myzobdella lugubris* now the species comprises 11 junior synonyms. Sawyer et al. (1975) included *Cystobranchus virginicus* as a junior synonym of *M. lugubris* based on a sample collected by Paperna & Zwerner (1974) York River, Virginia, USA. That sample resembles morphologically to *Myzobdella lugubris*, hence it could have been only a misidentification of samples by Paperna & Zwerner (1974). *Cystobranchus virginicus* sensu stricto has distinctive characters that separate it from *Myzobdella* species as distinct annulation (7 instead 14), testes in six pairs (instead five pairs) and present pulsatile vesicles (instead body surface smooth) (Hoffman, 1964). Thus, in our opinion, *C. virginicus* is a valid species, not junior synonyms of *M. lugubris*.

## Conclusions

External traits such as body color and number and shape of dorsal lines are a highly variable in *M. lugubris*. These, together with the broad salinity spectrum of habitat and the low host specificity, have confused taxonomists who described additional species repeatedly. Using molecular technics, especially comparison of DNA “barcoding” region, we identified all the samples as members of the same species, which seems to be widely introduced in Mexico, Central America and Europe. Our study highlights the need to include molecular data in order to evaluate morphological variation, together with a wide geographical sampling across the species distribution range.

## Data Availability

Voucher specimens were deposited in the Coleccción Nacional de Helmintos, Instituto de Biología, Universidad Nacional Autónoma de Mexico, Mexico City (collection numbers: 11660 and 11661). Newly generated DNA sequences are available in GenBank database at https://www.ncbi.nlm.nih.gov/genbank/ reference numbers indicated in Table 1.
